# Overexpression of Serotonin *N*-Acetyltransferase Cloned from the Green Algae *Chara braunii* Confers Peroxidizing Herbicide Tolerance Through the Induction of *Protoporphyrinogen Oxidase 1* in Rice

**DOI:** 10.3390/antiox15080942

**Published:** 2026-07-29

**Authors:** Kyungjin Lee, Kyoungwhan Back

**Affiliations:** Department of Molecular Biotechnology, College of Agriculture and Life Sciences, Chonnam National University, Gwangju 61186, Republic of Korea; nicekj7@hanmail.net

**Keywords:** butafenacil, cadmium, *Chara braunii*, melatonin, peroxidizing herbicide, serotonin *N*-acetyltransferase, transgenic rice

## Abstract

Serotonin *N*-acetyltransferase (SNAT) is pivotal to melatonin biosynthesis, catalyzing either serotonin into *N*-acetylserotonin or 5-methoxytryptamine (5-MT) into melatonin. Many *SNAT* genes have been cloned from a wide range of organisms, including animals and plants, but none has been reported in the model streptophyte green alga *Chara braunii*. Here, we found one archaeal CbSNAT homologue in the *C. braunii* genome, which showed 28% and 27% amino acid homology with human Naa50 and rice SNAT3, respectively. The *CbSNAT* gene, encoding a 172-amino acid protein, was expressed and purified in *Escherichia coli*. The recombinant CbSNAT protein exhibited SNAT enzyme activity toward serotonin (*K*_m_ = 293 μM) and 5-MT (*K*_m_ = 28 μM), and was located in the cytoplasm of tobacco cells, similar to other archaeal SNAT homolog proteins. To assess whether *CbSNAT* was functionally coupled to melatonin biosynthesis, *CbSNAT* was overexpressed in rice. Transgenic *CbSNAT*-overexpressing rice seedlings showed enhanced melatonin synthesis and resistance to both cadmium and the herbicide butafenacil. The cadmium resistance was due to the increased expression of chaperone genes such as *BIP3*, *BIP4*, and *BIP5*, whereas the butafenacil resistance resulted from increased expression of *protoporphyrinogen oxidase 1*, a target gene of butafenacil, via the transcriptional regulatory effects of melatonin. The discovery of a *CbSNAT* gene in *C. braunii* opens a new avenue for its use as a genetic resource in the development of crops exhibiting stress resistance to toxic compounds found in modern agriculture.

## 1. Introduction

Melatonin is a ubiquitous molecule with a diverse array of physiological functions that is found in all living organisms tested to date. In animals, melatonin is involved in the circadian rhythm, seasonal reproduction, redox homeostasis, innate immunity, anti-inflammation, anti-aging, and the sleep–wake cycle [1,2]. In plants, melatonin plays pleiotropic roles as either a potent antioxidant or signaling molecule regulating multifunctional roles under stressed and unstressed conditions. For instance, melatonin promotes seed germination and seedling growth by upregulating gibberellic acid synthesis and photosynthesis, respectively [3,4,5]. Moreover, the effect of melatonin treatment on seed germination and seedling growth is more prominent in plants exposed to stressors such as salt and drought [6,7]. Melatonin’s antistress effects originate from its direct free radical scavenging potential in bacteria such as Alphaproteobacteria and cyanobacteria, which later evolved into the mitochondria and chloroplasts of eukaryotes according to the endosymbiotic theory [1]. Thus, it is hypothesized that all cells, including mitochondria and chloroplasts, have the capacity to synthesize melatonin, which displays many physiological functions through melatonin receptor-dependent or -independent pathways [8]. Notably, a diverse array of melatonin’s physiological roles in plants are attributed to the production of melatonin metabolites, such as 2-hydroxymelatonin and 3-hydroxymelatonin, which trigger seed germination and seedling growth via reactive oxygen species (ROS) production [9,10].

In plants, melatonin biosynthesis starts with the aromatic amino acid tryptophan as the initial substrate, and requires four enzymatic reactions catalyzed by tryptophan decarboxylase (TDC), tryptamine 5-hydroxylase (T5H), serotonin *N*-acetyltransferase (SNAT; also called arylalkylamine *N*-acetyltransferase, AANAT), and *N*-acetylserotonin *O*-methyltransferase (ASMT) [11]. With the exception of *T5H*, all of these genes exist in plants as multiple gene families [11]. For example, rice possesses at least two *TDC*s, four *SNAT*s, and four *ASMT*s, including a caffeic acid *O*-methyltransferase, suggestive of multiple regulatory pathways for melatonin biosynthesis. Among these four genes, *SNAT* has gained attention as it is either the penultimate or ultimate step in synthesizing melatonin, depending on the substrates. As the penultimate enzyme, SNAT catalyzes *N*-acetylserotonin production using serotonin as a substrate; as the final enzyme, SNAT catalyzes 5-methoxytryptamine (5-MT) into melatonin.

Due to the dual substrate affinity of SNAT toward melatonin biosynthesis, attention has focused on cloning *SNAT* in various species, and it has long been used to engineer melatonin biosynthesis. The first transgenic tomato and tobacco plants overexpressing *SNAT* genes cloned from *Chlamydomonas reinhardtii* and human were reported in 2009; the transgenic plants showed elevated melatonin levels [12,13]. This was followed by transgenic rice plants overexpressing human *SNAT*, which showed cold resistance via melatonin overproduction [14]. Because of the absence of animal *SNAT* homologs in plants, the first *SNAT* gene in plants was cloned *de novo* from rice, followed by many other plant species and even cyanobacteria [11,15].

Butafenacil is a diphenyl ether herbicide (DPE) that exhibits herbicidal activity with a photodynamic mode by inhibiting protoporphyrinogen oxidase (PPO), which catalyzes the oxidation of protoporphyrinogen IX (protogen IX) into protoporphyrin (proto IX) as the penultimate enzyme at the branch point for the biosynthetic pathways of chlorophylls and hemes. Butafenacil-induced PPO inhibition paradoxically yields proto IX in cells as an enzymatic product. This accumulation of proto IX leads to the production of singlet oxygen and provokes membrane lipid peroxidation, causing cellular death via a light-dependent mechanism [16]. Therefore, overexpression of the *PPO* gene is a key strategy for generating plants resistant to butafenacil [17]. Additionally, the overexpression of antioxidant and glutathione transferase genes can confer DPE herbicide resistance through the scavenging of ROS and the binding of glutathione to DPE herbicides, respectively [18,19]. However, there are no reports on whether melatonin induces the *PPO* gene to cause resistance to DPE herbicides.

*Chara braunii* is a multicellular plant-like green alga species in the family Characeae. As the closest living relative of plants, *C. braunii* is a model organism for the study of the evolution and colonization of land plants. Its whole genome was first sequenced in 2018, which revealed that it synthesizes many phytohormones such as auxin, cytokinin, ethylene, abscisic acid, and strigolactone [20]. *C. braunii* displays a defense response similar to that of plants against abiotic stressors such as salt [21]. In addition to the abovementioned phytohormones, melatonin was identified at concentrations of 4 ng/g fresh weight during the day and 400 ng/g fresh weight in the dark, revealing a circadian rhythm of melatonin in *Chara australis* [22]. It has also been reported that exogenous melatonin treatment increases photosynthesis in *C. australis* [23] as with land plants [24,25]. However, melatonin biosynthesis genes have not been cloned in *Chara* species. Specifically, even though *SNAT* genes have been cloned from a wide range of plants and animals, no *SNAT* homolog of previously discovered *SNAT* genes has been found in the genome of *C. braunii*. Recently, a novel class of *SNAT* genes was cloned from the archaeon *Thermoplasma volcanium*, which belongs to the protein *N*-acetyltransferase family [26]. Subsequently, a homolog of this archaeal *SNAT* gene (*CbSNAT*) was found in the genome of *C. braunii*.

The goal of this study is to express candidate *CbSNAT* gene in *Escherichia coli* to purify recombinant CbSNAT proteins and to characterize the enzymatic properties of SNAT in vitro. Additionally, we intend to investigate the effects of ectopic overexpression of *CbSNAT* on in vivo melatonin biosynthesis in rice and phenotypic changes in response to abiotic stress. Here, a candidate *CbSNAT* as an archaeal *SNAT* homolog was chemically synthesized and expressed in *E. coli*, followed by affinity purification. The purified recombinant CbSNAT protein exhibited SNAT enzyme activity. Further functional integrity of *CbSNAT* was assessed through the heterologous overexpression of *CbSNAT* in rice. The resulting *CbSNAT*-overexpressing rice exhibited enhanced melatonin synthesis and tolerance against the peroxidizing herbicide butafenacil, which kills plants via increased ROS production [17].

## 2. Materials and Methods

### 2.1. Chemical Synthesis of C. braunii SNAT Nucleotides

Based on the nucleotide sequence information of *CbSNAT* (GenBank accession number GBG84821), a 519 bp long full-length *CbSNAT* nucleotide encoding 172 amino acids was custom synthesized (Bioneer, Daejeon, South Korea).

### 2.2. Escherichia coli Expression and Affinity Purification of Recombinant CbSNAT Proteins

In order to achieve a recombinant CbSNAT protein, *Escherichia coli* expression system was employed as described previously elsewhere [10,26]. Briefly, the full-length synthetic *CbSNAT* gene was amplified by polymerase chain reaction (PCR) by using primer set harboring 5′ *attB* sequence (*CbSNAT* forward primer, 5′-AAA AAG CAG GCT CCA TGG ACA CGT TTT CGG TC-3′; *CbSNAT* reverse primer, 5′-AGA AAG CTG GGT AAG AGG TAA TGA CAG CTT-3′) with a template plasmid containing the synthetic *CbSNAT* DNA (pBHA-CbSNAT) which was provided by Bioneer (Daejeon, South Korea). The resulting PCR product was further amplified by PCR using an *attB* primer set (*attB* forward primer, 5′-GGG GAC AAG TTT GTA CAA AAA AGC AGG CT-3′; *attB* reverse primer, 5′-GGG GAC CAC TTT GTA CAA GAA AGC TGG GT-3′). The full-length *CbSNAT* PCR product was gel-purified and recombined into the pDONR221 Gateway vector (Invitrogen, Carlsbad, CA, USA) via the BP recombination reaction. The resulting pDONR221-CbSNAT entry vector was then recombined with the pET60 destination vector (Invitrogen) via LR recombination to yield pET60-CbSNAT. As for a pET32b-CbSNAT plasmid construction, pDONR221-CbSNAT was digested with *Nco*I and *Eco*RV, and the *CbSNAT* insert was ligated into pET32b, which had been previously digested with the same restriction enzymes, to generate pET32b-CbSNAT. Both pET60-CbSNAT and pET32b-CbSNAT plasmids were transformed into *E. coli* strain BL21(DE3) (Invitrogen). Additional *E. coli* culture, isopropyl-β-D-thiogalactopyranoside (IPTG; Sigma, St. Louis, MO, USA) treatment, and affinity (Ni^2+^) purification were performed according to the user manual provided by Invitrogen. The purified recombinant CbSNAT proteins were mixed with the equal volume of glycerol and stored at −80 °C until use. Protein concentrations were determined using the Bradford method and a protein assay dye (Bio-Rad, Hercules, CA, USA).

### 2.3. Homology and Phylogenetic Analysis

The analysis of amino acid sequence homology search using rice SNAT3 (OsSNAT3) and human Naa50 as query sequences were carried out with the BLASTp tool in the non-redundant protein sequences databases at the National Center for Biotechnology Information (NCBI; http://www.ncbi.nlm.nih.gov/, accessed on 22 February 2021). The BLAST-Explorer program (version 2, Information Genomique & Structurale, Marseille, France) was utilized for perform phylogenetic tree and multiple sequence analyses [27,28].

### 2.4. Enzymatic Assays for SNAT

As for in vitro SNAT enzymatic assay, the purified recombinant GST-CbSNAT (1 µg) was employed and performed in a 100 µL final volume containing 0.5 mM serotonin (or other substrates), and 0.5 mM acetyl-CoA in 100 mM potassium phosphate (pH 7.8) at 55 °C for 30 min. The resulting reaction was terminated with the addition of 20 µL acetic acid and 30 µL methanol of which ten microliters of enzymatic reaction was analyzed by reverse phase high-performance liquid chromatography (HPLC) (Waters, Milford, MA, USA) using a SunFire C18 column (Waters; 4.6 × 150 mm) eluted using an isocratic profile of 42% (*v*/*v*) methanol with 0.3% [*v*/*v*] trifluoroacetic acid) at a flow rate of 0.45 mL/min. Compounds were detected at 280 nm (excitation) and 348 nm (emission). The substrate affinity (*K*_m_) and maximum reaction rate (*V*_max_) were calculated using Lineweaver–Burk plots. All analyses were performed in triplicate.

### 2.5. Subcellular Localization Analysis of CbSNAT

The tobacco transient expression system was used to investigate the subcellular location of CbSNAT protein. The pER-mCherry binary vector kindly provided by Dr. H.G. Kang (Texas State University, San Marcos, TX, USA). First of all, the full-length of *CbSNAT* cDNA was amplified by PCR using a primer set containing *Asc*I sites (*Asc*I forward primer 5′-GGC GCG CCA TGG ACA CGT TTT TGG TC-3′; *Asc*I reverse primer, 5′-GGC GCG CCG AGA GGT AAT GAC AGC TTC-3′) with a template plasmid pBHA-CbSNAT. The resulting *CbSNAT* PCR product was initially ligated into the TA vector (RBC Bioscience, New Taipei City, Taiwan) followed by *Asc*I restriction endonuclease enzyme digestion. The resulting *Asc*I insert of *CbSNAT* was then cloned into the *Asc*I site of the binary vector pER8-mCherry containing the estrogen-inducible XVE promoter (Pxve) resulting in pER8-CbSNAT-mCherry. The pER8-CbSNAT-mCherry plasmid construct was transformed into the *Agrobacterium tumefaciens* GV2260 strain using the freeze–thaw method. Agrobacterium-mediated transient expression of CbSNAT-mCherry fusion protein and confocal microscope analysis (TCS-SP5; Leica, Wetzlar, Germany) were performed as described previously [10].

### 2.6. Agrobacterium-Mediated Transformation for Developing Transgenic Rice Overexpressing CbSNAT

The ectopic overexpression of *CbSNAT* into rice nuclear genome was accomplished by using a gateway binary vector pIPKb002. The pIPKb002 binary vector was kindly provided by Dr. J. Kumlehn (Leibniz Institute of Plant Genetics and Crop Plant Search, Gatersleben, Germany) [29]. Briefly, the pDONR221-CbSNAT entry vector was recombined with the pIPKb002 destination vector via LR recombination to yield pIPKb002-CbSNAT in which the *CbSNAT* gene is expressed under the constitutive maize ubiquitin promoter. The pIPKb002-CbSNAT binary vector was transformed into *Agrobacterium tumefaciens* strain LBA4404. As previously described [26], transgenic rice plants were produced through somatic embryogenesis using *Agrobacterium*-mediated rice transformation using callus produced from the Japonica rice variety Dongjin.

### 2.7. Melatonin Quantification by HPLC Analysis

Rice seedlings were harvested and frozen in liquid nitrogen; leaf samples (0.1 g) were pulverized to a powder in liquid nitrogen using the TissueLyser II (Qiagen, Tokyo, Japan). The sample powders were extracted with 1 mL chloroform followed by high-performance liquid chromatography (HPLC) with a fluorescence detector system (Waters, Milford, MA) as described previously [10].

### 2.8. Cadmium and Herbicide Treatment in the CbSNAT-Overexpressing Transgenic Rice Plants

The 7-day-old seedlings collected from Murashige and Skoog (MS) medium were incubated in 50 mL polypropylene conical tubes containing 30 mL water containing 0.5 mM cadmium or 0.1 µM butafenacil (a kind gift from professor Guh (Chonnam National University, Gwangju, Republic of Korea)) and incubated for 3 days and 40 h under a continuous light and a 14 h light/10 h dark photoperiod condition (60 µmol m^−2^ s^−1^) at 28 °C/24 °C (day/night), respectively. The levels of malondialdehyde (MDA) were measured at wavelengths of 440, 532, and 600 nm using a spectrophotometer (Optizen POP-Bio; Mecasys, Daejeon, Republic of Korea) as described previously [26]. Chlorophyll contents were measured as follows: the rice leaf powder (100 mg) was extracted with 1 mL of 0.1 M NH_4_OH (containing 100% methanol). Chlorophyll concentrations were determined at wavelengths of 647, 664, and 750 nm using a spectrophotometer (Optizen POP-Bio) as described previously [30].

### 2.9. Total RNA Isolation and Quantitative–Polymerase Chain Reaction (qPCR)

Total RNA isolation, first-strand cDNA, and qPCR for monitoring genes involved in oxidative stress were followed as described previously [26]. The sequences of primers were listed previously [31]. The sequences of forward and reverse primers of *PPO1* were 5′-GTG ATA TCT TGC GGC CAC TT-3′ and 5′-GGA GCA CGA TTT GGA AAG AG-3′, respectively. The *PPO2* primers used in this study were a forward primer (5′-GGT GCC ATC TTG TCC AAA CT-3′) and a reverse primer (5′-ACT CCA TCA CAG CAA CAT GC-3′).

### 2.10. Statistical Analysis

Prior to statistical analysis, data were evaluated for normality and homogeneity of variance using the Shapiro–Wilk test and Levene’s test, respectively. Since the data satisfied both assumptions, a one-way analysis of variance (ANOVA) was performed to determine the significant differences among the treatment groups (IBM Corp., Armonk, NY, USA). Following the ANOVA, Tukey’s Honestly Significant Difference (HSD) test was applied as a post hoc analysis for multiple comparisons. Mean values, indicated by different letters, represent statistically significant differences at the *p* < 0.05. Data are presented as mean ± standard deviation from at least three independent experimental replicates.

## 3. Results

### 3.1. BLASTp Search and Chemical Synthesis of CbSNAT

**Figure 1:**
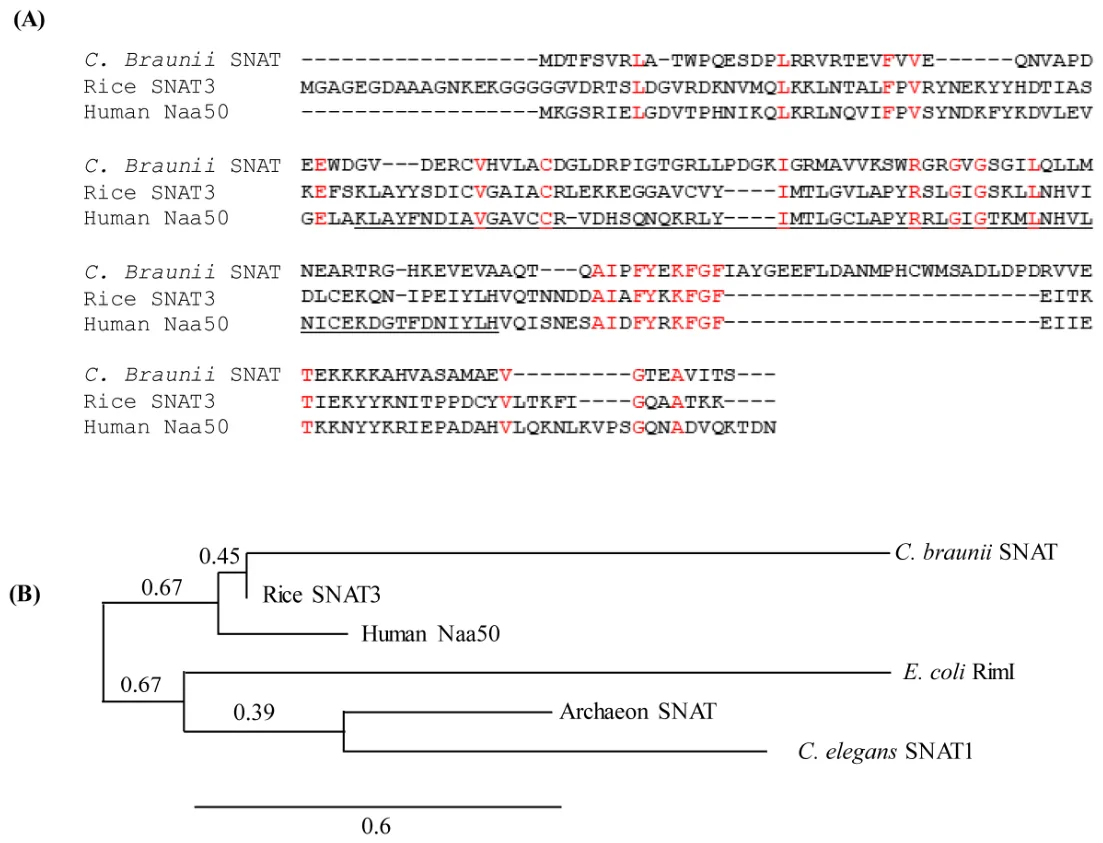
(**A**) Amino acid sequence comparison among *Chara braunii* SNAT, rice SNAT3, and human Naa50. The acetyl coenzyme A binding sites are underlined. Dashes denote gaps. Conserved amino acids are denoted as red letters. (**B**) Phylogenetic tree of *C. braunii SNAT* gene and archaeal *SNAT* homolog genes. The scale bar represents 0.6 substitutions per site. GenBank accession numbers are archaeon *SNAT* (NC_003413), *Caenorhabditis elegans SNAT1* (NM_065818), *E. coli RimI* (WP_137442509), *C. braunii SNAT* (GBG84821), *rice SNAT3* (AK241100), and human *Naa50* (BAB14397).

The BLASTp program (https://blast.ncbi.nlm.nih.gov/Blast.cgi, accessed on 22 February 2021) indicated that CbSNAT (GenBank accession number GBG84821) encodes 172 amino acids (aa), with 28% identity and 52% positivity to human Naa50, with a 30% query cover value (Figure 1). The candidate CbSNAT exhibited 27% aa identity and 46% positivity to rice SNAT3 with a 62% query cover value. Although there were low aa homologies within a short region of protein among CbSNAT, human Naa50, and rice SNAT3, CbSNAT is the sole SNAT candidate to date in *C. braunii* genome showing aa sequence homology to SNAT proteins. Phylogenic analysis placed CbSNAT in the archaeal SNAT clade, in which CbSNAT appears to be the closest homolog of rice SNAT3 (Figure 1B). Archaeal SNAT-related homologs include *Caenorhabditis elegans* SNAT, human Naa50, and *E. coli* RimI, and possess SNAT enzyme activity [26].

The reported nucleotide sequence was chemically synthesized without altering the codon usage. This was because the G+C content of natural CbSNAT was 61%, higher than that of rice SNAT3 (GenBank access number AK241100) at 51%. Due to the high G+C content of *CbSNAT*, polyadenylation signals (AAUAAA) and mRNA destabilization sequences (AUUUA), which can commonly be caused by a high A+T content, did not occur. In general, the presence of AAUAAA and AUUUA sequences inhibits ectopic overexpression in host cells [32]. The synthesized *CbSNAT* nucleotides were used in studies of *E. coli* expression and ectopic overexpression in the rice genome.

### 3.2. Purification of Recombinant CbSNAT Proteins and Enzyme Kinetic Analysis

**Figure 2:**
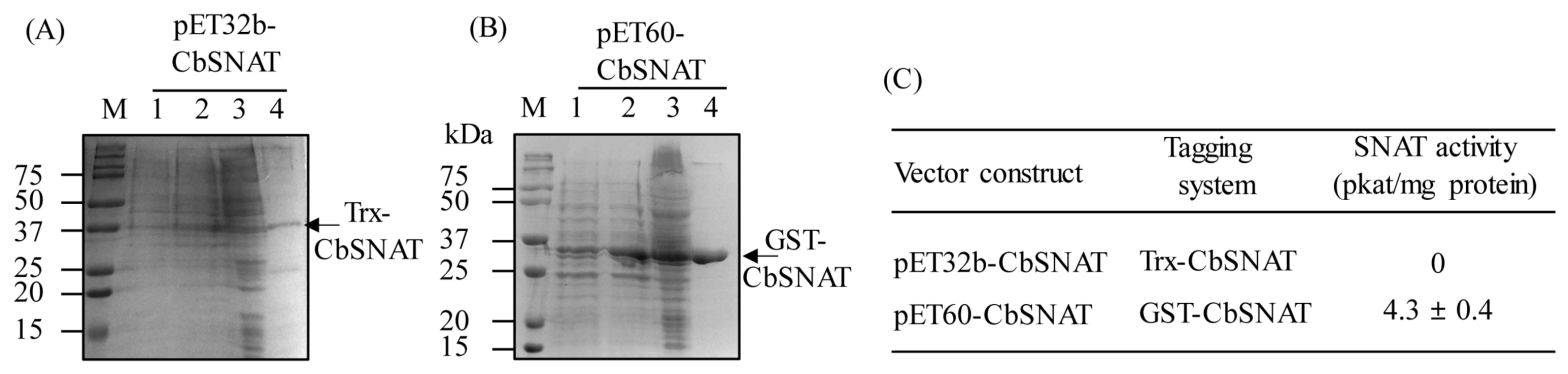
Heterologous overexpression of *C. braunii SNAT* (*CbSNAT*) in *Escherichia coli*, affinity purification of CbSNAT recombinant proteins, and their SNAT enzymatic activities. (**A**) Expression and affinity purification of CbSNAT as a thioredoxin (Trx) fusion protein using a pET32b vector. (**B**) Expression and affinity purification of CbSNAT as a gluthathione (GST) fusion protein using a pET60 vector. Arrows indicate the recombinant proteins. (**C**) Measurement of SNAT enzyme activity (SNAT) using serotonin as a substrate. *E. coli* BL21 (DE3) cells harboring plasmids were induced with isopropyl β-d-1-thiogalactopyranoside (IPTG) for 5 h at 24 °C. M, molecular size marker; lane 1, total proteins in 20 µL aliquots of bacterial culture without IPTG; lane 2, total proteins in 15 µL aliquots of bacterial culture with IPTG; lane 3, 20 µg soluble protein; lane 4, 5 µg recombinant proteins purified by affinity chromatography. Protein samples were separated using 12% SDS-PAGE and stained with Coomassie blue.

**Figure 3:**
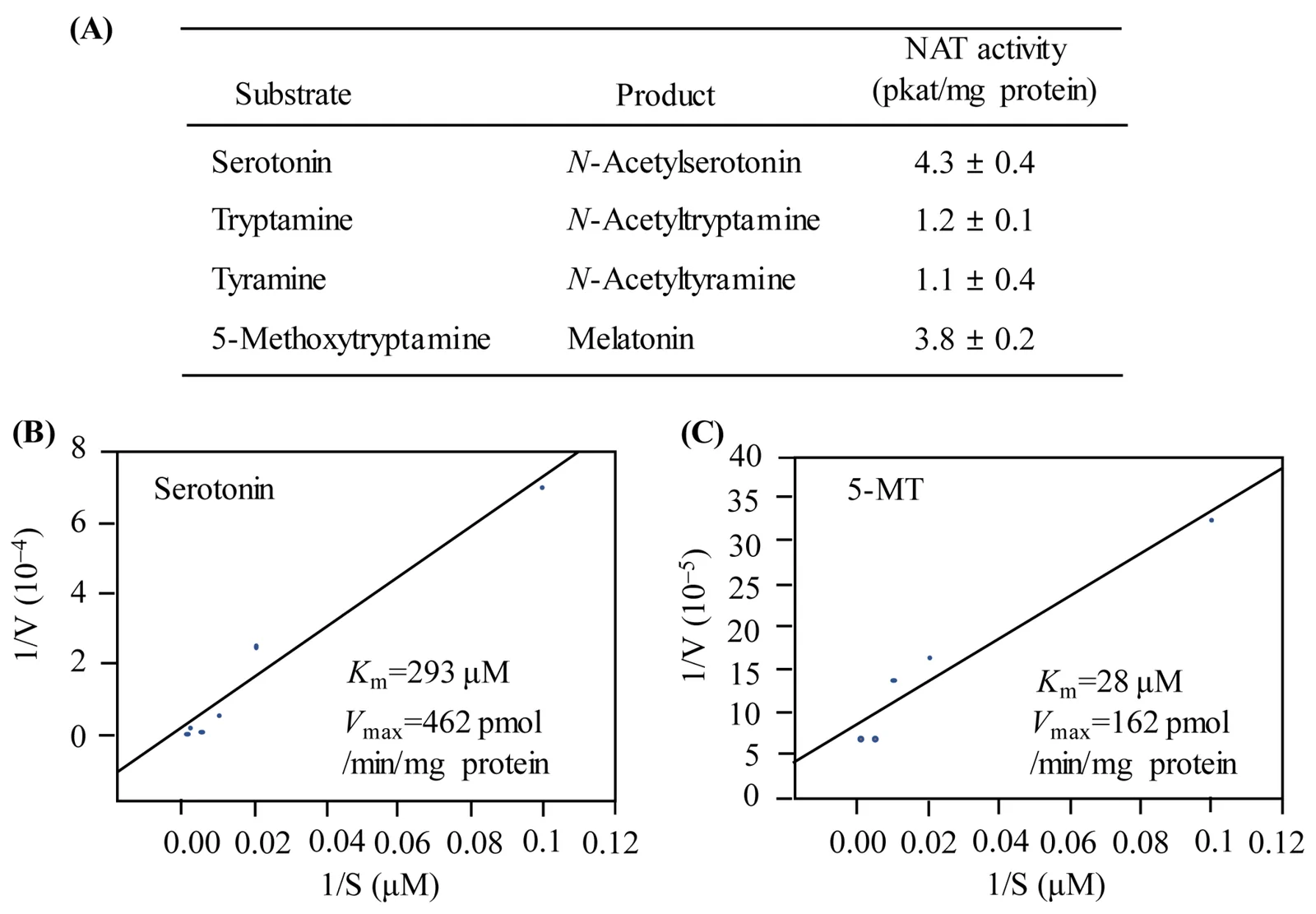
SNAT enzyme kinetic analysis of purified recombinant GST-CbSNAT. (**A**) Serotonin *N*-acetyltransferase enzyme activity on various arylalkylamine substrates. (**B**) *K*_m_ and *V*_max_ values for serotonin substrate. (**C**) *K*_m_ and *V*_max_ values for 5-methoxytryptamine (5-MT) substrate. The recombinant purified GST-CbSNAT (1 µg) was assayed for 0.5 h at 55 °C at pH 7.8 in the presence of 0.5 mM various substrates and 0.5 mM acetyl CoA followed by high-performance liquid chromatography HPLC detection. Values are means ± SD (*n* = 3).

The synthetic full-length *CbSNAT* gene was first cloned into pET300 to express CbSNAT in an N-terminal hexa-histidine tagged form (His6-CbSNAT), followed by Ni^2+^ affinity purification in *E. coli*. Unfortunately, the resulting recombinant protein could not be purified, possibly due to the insoluble expression of CbSNAT in *E. coli*. Thus, N-terminal fusion tags using thioredoxin (Trx) or glutathione S-transferase (GST) were introduced to enhance CbSNAT soluble expression in *E. coli* [26,33]. GST-CbSNAT exhibited higher solubility than Trx-CbSNAT based on Coomassie blue staining (compare lane 3 between Figure 2A,B). Purified recombinant Trx-CbSNAT did not show SNAT enzyme activity, whereas GST-CbSNAT showed SNAT activity of 3.8 pkat/mg protein using serotonin as a substrate. Given that all SNAT enzymes from animals and plants can accept many other amines as substrates [26,34,35], substrate specificity analyses were conducted on the amines serotonin, tryptamine, tyramine, and 5-MT. The highest SNAT enzyme activity was observed using serotonin as a substrate, followed by 5-MT, tryptamine, and tyramine (Figure 3A). The fact that CbSNAT showed the lowest level of SNAT activity toward tyramine was in line with CeSNAT1 [26]. In contrast to CbSNAT, 5-MT exhibited the highest SNAT enzyme activity in CeSNAT1.

**Figure 4:**
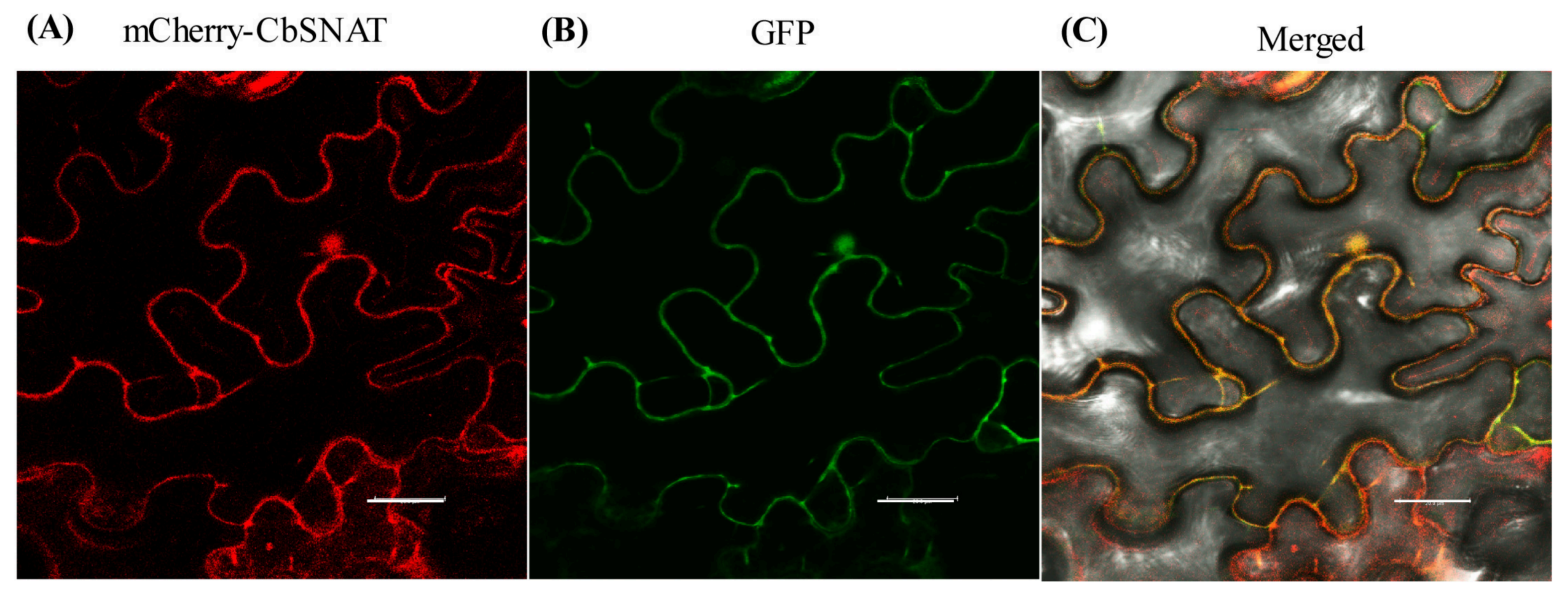
Localization of CbSNAT. (**A**) Red fluorescence of CbSNAT-mCherry of *Agrobacterium*-infiltrated tobacco. (**B**) Green fluorescence of cytoplasmic green fluorescent protein (GFP). (**C**) The two fluorescence images were merged (**A**,**B**). The leaves of tobacco (*Nicotiana benthamiana*), a native Australian species, were infiltrated with *Agrobacterium* harboring the XVE-inducible *CbSNAT*-mCherry binary vector and were grown for two days in a growth room before XVE-induction and visualization using confocal microscopy. Bars = 20 μm.

The substrate affinity for serotonin and 5-MT were measured using Lineweaver–Burk plots (Figure 3B,C). The Michaelis–Menten (*K*_m_) values for serotonin and 5-MT were 293 μM and 28 μM, respectively. The catalytic efficiency (*V*_max_/*K*_m_) was 3.7 times higher for 5-MT than for serotonin. Similar results were observed in CeSNAT1 and rice SNAT3 [26]. In summary, *CbSNAT* is a true *SNAT* found in the *C. braunii* genome and is a homologous gene of archaeal *SNAT*. In contrast to rice SNAT1 and rice SNAT2, which are located in chloroplasts, archaeal SNAT, human Naa50, and rice SNAT3 are located in the cytoplasm [26]. To determine which organelles CbSNAT is expressed in, an in silico TargetP analysis [36] was performed; the absence of either chloroplast or mitochondrial transport peptides indicated that CbSNAT is a cytoplasmic protein. As predicted by the TargetP analysis, CbSNAT exhibited strong red fluorescence overlapping with cytoplasmic GFP protein, confirming its cytoplasmic location (Figure 4).

### 3.3. Cadmium Tolerance of CbSNAT-Overexpressing Transgenic Rice Plants

**Figure 5:**
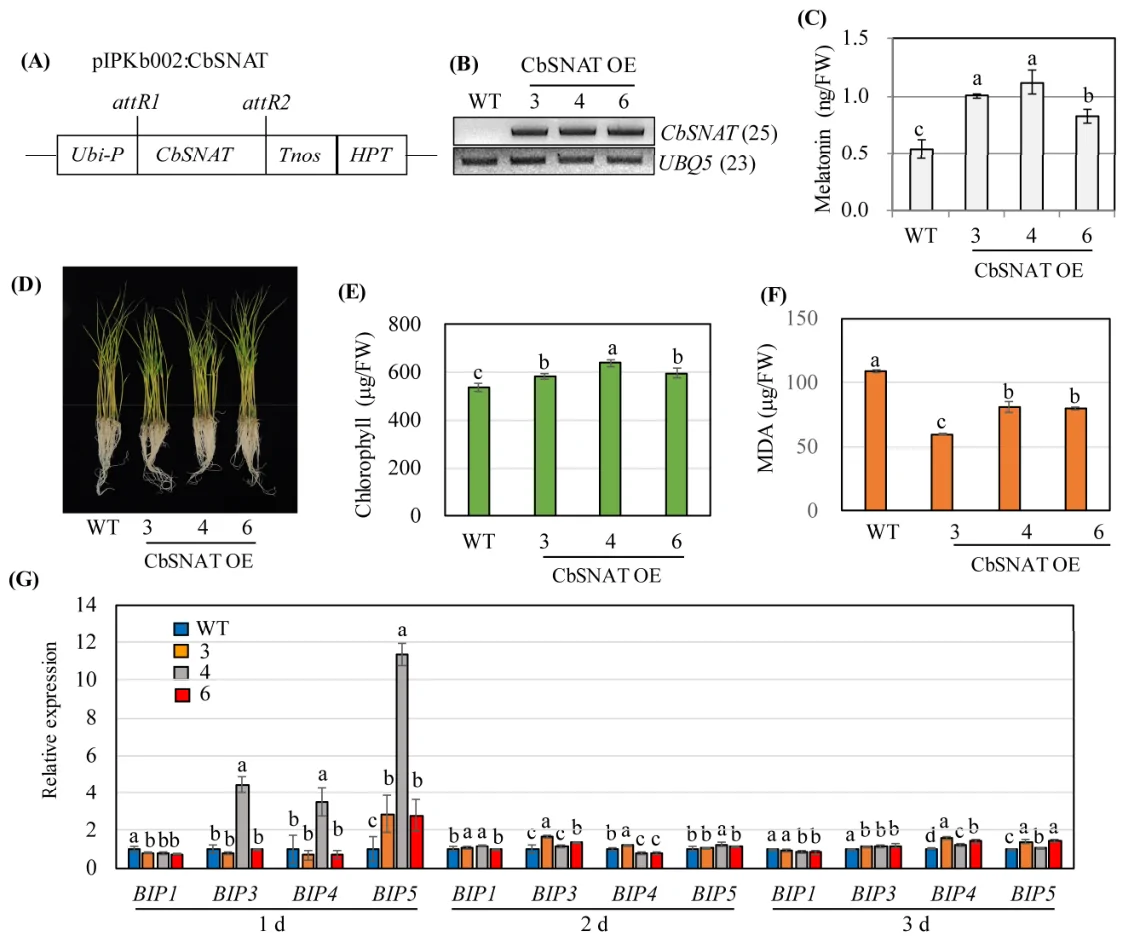
Cadmium tolerance of transgenic rice plants overexpressing *CbSNAT*. (**A**) Schematic diagram of *T-DNA* region in pIPKb002:CbSNAT binary vector. (**B**) Expression of *CbSNAT* by RT-PCR analyses of the *CbSNAT* overexpression lines (CbSNAT OE) and wild type (WT) in 7-day-old rice seedlings. (**C**) Quantification of melatonin. (**D**) Photograph of 7-day-old seedlings after 0.5 mM cadmium treatment for 3 days. (**E**) Measurement of chlorophyll levels. (**F**) Measurement of malondialdehyde (MDA) contents. (**G**) Time course of relative expression levels of chaperone-related *binding protein* (*BIP*) genes in response to cadmium treatment. *Ubi-P*, *maize ubiquitin promoter*; *CbSNAT*, *C. braunii SNAT*; *Tnos*, *nopaline synthase terminator*; *HPT*, *hygromycin phosphotransferase*; WT, wild type; *UBQ5*, *rice ubiquitin 5 gene*; CbSNAT OE, *CbSNAT*-overexpressing transgenic lines. GenBank accession number of *UBQ5* is AK061988. Different letters indicate significant differences among lines (n = 3; Tukey’s HSD; *p* < 0.05).

To assess whether *CbSNAT* is functionally coupled to melatonin biosynthesis in vivo, *CbSNAT* was overexpressed in rice under the control of the constitutive maize ubiquitin promoter using an *Agrobacterium*-mediated transformation system (Figure 5A). From the initial selection of 18 independent T_0_ transgenic lines surviving antibiotic hygromycin selection, six independent T_1_ transgenic rice seeds that exhibited an approximately 3:1 segregation of hygromycin resistance to non-resistance were selected. From these, three homozygous lines of T_2_ seeds showing 100% resistance to hygromycin were further selected to examine the effects of *CbSNAT* overexpression in rice seedlings. First, total RNA was extracted from 7-day-old rice seedlings and reverse-transcription polymerase chain reaction (RT-PCR) analysis was performed to assess *CbSNAT* overexpression. No detectable *CbSNAT* PCR product was observed in the wild type (WT), but high levels of *CbSNAT* expression were detected in all three transgenic lines (CbSNAT OE) (Figure 5B). The transgenic lines showed similar relative expression levels of *CbSNAT*, suggesting a marginal position effect of the *CbSNAT* transgene in the rice genome.

To determine whether *CbSNAT* overexpression is functionally associated with melatonin synthesis, melatonin levels were measured in 7-day-old CbSNAT OE seedlings using high-performance liquid chromatography. The three CbSNAT OE lines produced melatonin at an approximately two-fold-greater rate relative to the WT (Figure 5C), indicating a close positive correlation between *CbSNAT* overexpression and endogenous melatonin levels.

Since melatonin is positively involved in cadmium tolerance in plants [37,38], CbSNAT OE seedlings were challenged with 0.5 mM cadmium treatment for 3 days as described previously [31,37] (Figure 5D). Following cadmium exposure, the seedlings of CbSNAT OE lines contained higher chlorophyll contents than the WT (Figure 5E). Similar to chlorophyll contents, the levels of malondialdehyde (MDA), an end product of lipid peroxidation, were reduced in the CbSNAT OE lines compared to those of the WT, indicating that the CbSNAT OE lines exhibited cadmium tolerance (Figure 5F).

Based on a report indicating significant roles of chaperone-related genes (rather than antioxidant-related genes) in rice *SNAT3*-mediated cadmium tolerance [31], the expression levels of *binding protein*-encoding genes (*BIP*) were evaluated in the CbSNAT OE lines. In the CbSNAT OE lines, *BIP5* expression levels were significantly upregulated, and *BIP1* downregulated, compared to the WT at 1 d after cadmium treatment (Figure 5G). At 3 d after cadmium treatment, *BIP3*, *BIP4* and *BIP5* expression was higher in the CbSNAT OE lines than that of the WT. The enhanced expression of *BIP* genes in the CbSNAT OE lines was similar to the mechanism of rice *SNAT3*-mediated cadmium tolerance [31]. Overall, these data revealed that the CbSNAT OE lines had increased melatonin levels, resulting in enhanced tolerance to cadmium stress.

### 3.4. Butafenacil Tolerance of CbSNAT-Overexpressing Transgenic Rice Plants

**Figure 6:**
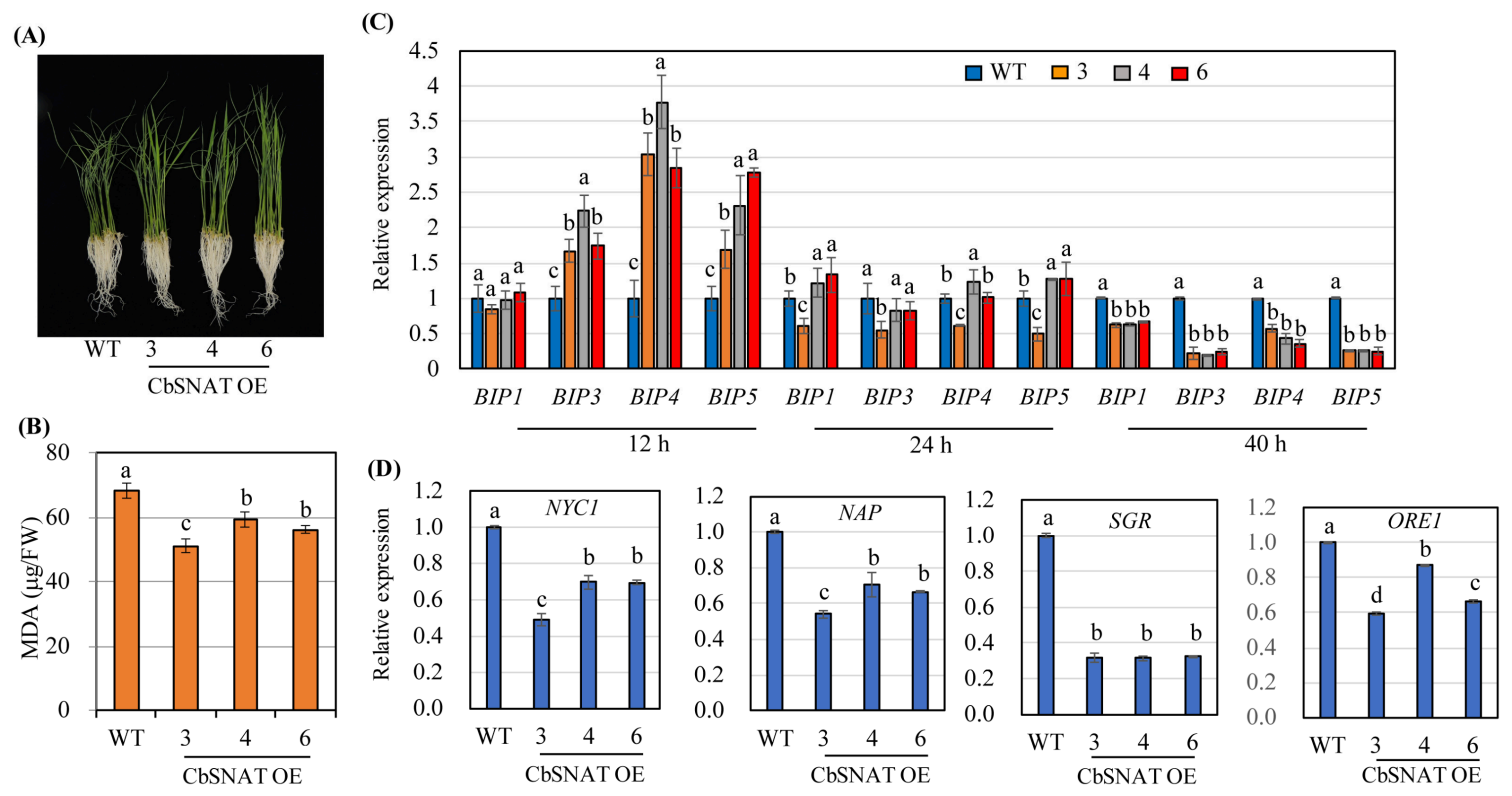
Herbicide butafenacil tolerance of transgenic rice plants overexpressing *CbSNAT*. (**A**) Photograph of 7-day-old seedlings after 0.1 µM butafenacil treatment for 40 h. (**B**) Measurement of malondialdehyde (MDA) contents in CbSNAT OE and WT. (**C**) Time course of relative expression levels of chaperone-related *binding protein* (*BIP*) genes in response to butafenacil treatment. (**D**) Relative expression levels of senescence-related genes. CbSNAT OE, *CbSNAT*-overexpressing transgenic lines. Different letters indicate significant differences among lines (n = 3; Tukey’s HSD; *p* < 0.05).

Due to the potent antioxidant activity of melatonin and melatonin-mediated induction of various antioxidant enzymes, melatonin can protect plants from various environmental stresses that would otherwise trigger ROS production and result in adverse effects on plant growth and development [11,37]. Butafenacil is a peroxidizing herbicide that causes ROS production in plants, thus leading to plant death [16]. To determine whether the CbSNAT OE lines exhibited resistance to butafenacil treatment, 0.1 μM butafenacil was applied to the roots of 7-day-old rice seedlings and cultured for 40 h under a 14 h light/10 h dark condition (Figure 6A). The CbSNAT OE lines had lower MDA levels than the WT (Figure 6B), suggesting that the CbSNAT OE lines had resistance to oxidative stress, as high MDA concentrations indicate increased oxidative damage.

To determine whether the oxidative stress tolerance of the CbSNAT OE lines was associated with increased *BIP* expression, as observed in cadmium tolerance, the *BIP* expression levels of 7-day-old rice seedlings treated with butafenacil were investigated. Contrary to the cadmium treatment results, the CbSNAT OE lines showed lower expression levels of all *BIP* genes compared to the WT (Figure 6C) at 40 h after butafenacil treatment, but *BIP3*, *BIP4*, and *BIP5* were transiently induced at 12 h after butafenacil treatment in the CbSNAT OE lines relative over the WT, suggesting that *BIP* expression was initially associated with butafenacil tolerance in the CbSNAT OE lines.

**Figure 7:**
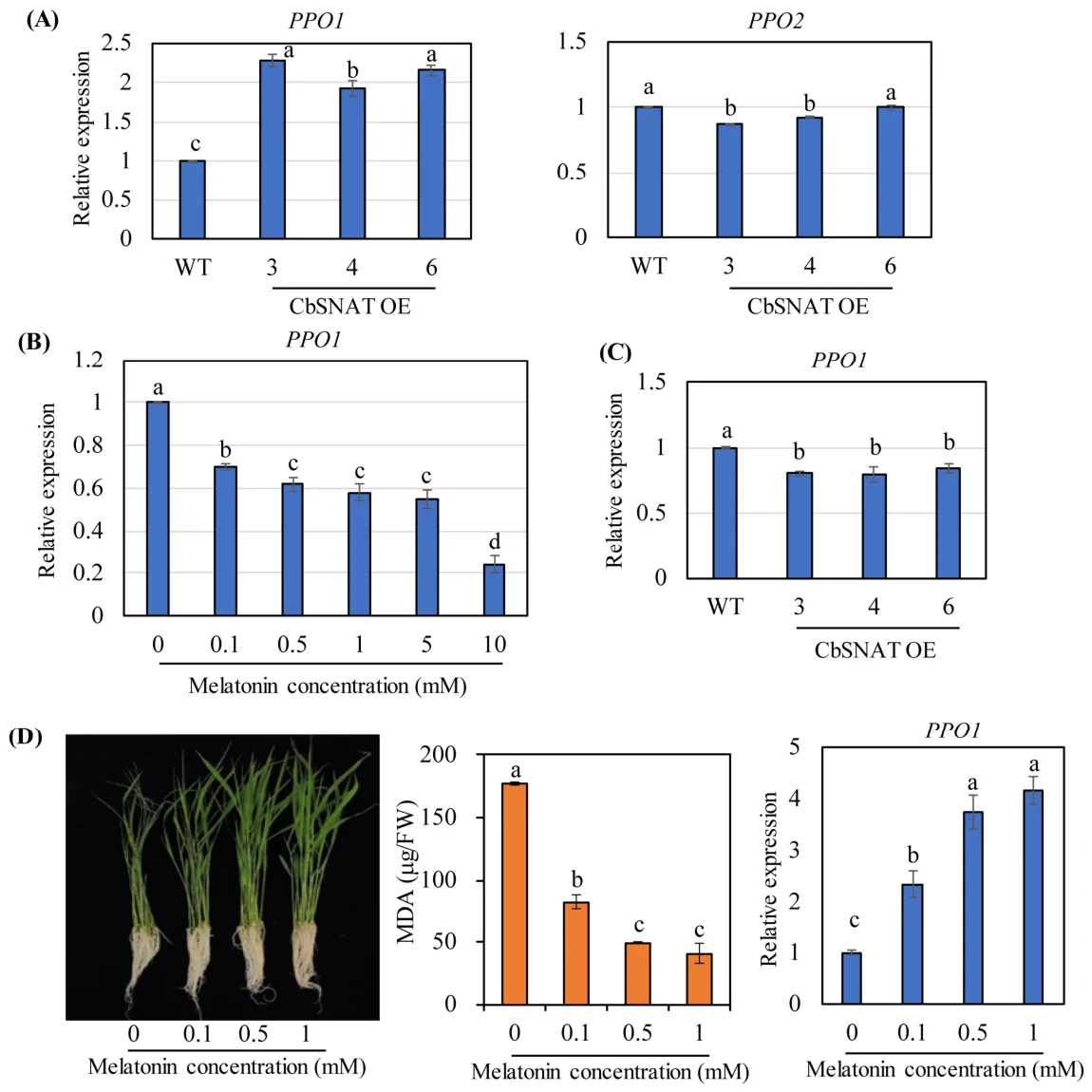
Expression levels of *protoporphyrinogen oxidases* (*PPO1* and *PPO2*) and butafenacil tolerance by exogenous melatonin treatment. (**A**) Relative expression levels of *PPO1* and *PPO2* genes after 0.1 µM butafenacil treatment for 40 h in the CbSNAT OE lines and WT. (**B**) Relative expression levels of *PPO1* in response to exogenous melatonin treatments in the 7-day-old WT seedlings for 24 h. (**C**) Relative expression levels of *PPO1* in the CbSNAT OE lines and WT seedlings without butafenacil treatment. (**D**) Exogenous melatonin treatment toward butafenacil tolerance and *PPO1* expression. Different letters indicate significant differences among lines (n = 3; Tukey’s HSD; *p* < 0.05).

Butafenacil resistance in the CbSNAT OE lines was further confirmed based on the expression levels of various senescence (or oxidative stress)-related genes such as *NON-YELLOW COLORING 1* (*NYC1*), *NAC transcription factor* (*NAP*), *STAY-GREEN* (*SGR*), and *ORESARA1* (*ORE1*) [39,40]. These senescence-related marker genes were downregulated in the CbSNAT OE lines compared to the WT, indicative of butafenacil resistance in the CbSNAT OE lines. To obtain more direct evidence of butafenacil resistance in the CbSNAT OE lines, we measured the expression levels of *PPO*, a butafenacil target gene. Two *PPO* genes exist in the plant nuclear genome: the chloroplast enzyme PPO1, which has an important role in butafenacil resistance [17], and the mitochondrial enzyme PPO2. After treatment with butafenacil, *PPO1* expression increased significantly in the CbSNAT OE lines compared to the WT, whereas *PPO2* showed similar expression levels in the CbSNAT OE lines and the WT (Figure 7A). Thus, an increase in *PPO1* appeared to be the primary driver of butafenacil resistance in the CbSNAT OE lines. To further confirm the increase in *PPO1* induced by melatonin, we investigated other transgenic rice plants overexpressing the *Stylonychia lemnae SNAT* (*SlSNAT*) and the rice *SNAT3* (*OsSNAT3*) [26,31]. Both SlSNAT OE and OsSNAT OE rice plants showed improved butafenacil resistance along with an increase in *PPO1* (Supplementary Figure S1).

This finding prompted us to investigate whether exogenous treatment with melatonin induced *PPO1* expression levels in rice seedlings. However, exogenous melatonin treatment showed the opposite result, suppressing *PPO1* expression levels in a dose-dependent manner (Figure 7B). Similar to the results of exogenous melatonin treatment, *PPO1* levels were lower in the CbSNAT OE lines than in the WT in the absence of butafenacil (Figure 7C). To obtain direct evidence of melatonin-mediated butafenacil resistance, rice seedlings were treated with melatonin for 24 h followed by butafenacil treatment for 40 h. As can be seen in Figure 7D, exogenous melatonin treatment conferred butafenacil resistance along with an increase in *PPO1*. The increase in *PPO1* expression in CbSNAT OE lines is due to the transcriptional regulatory effect of melatonin rather than its direct antioxidant effect on oxidative stress caused by butafenacil treatment. This reprogramming of gene expression by melatonin has been frequently reported in other studies as well [41,42,43,44,45].

## 4. Discussion

*SNAT* plays a key regulatory role in melatonin biosynthesis in all living organisms. Many *SNAT* genes have been cloned and functionally characterized from animals, plants, fungi, and bacteria [11,26,31]. Because *SNATs* play an essential role in melatonin biosynthesis, *SNATs* have been adopted as one of the key target genes to increase or decrease melatonin levels through ectopic overexpression and RNA interference (RNAi) techniques, respectively [46,47,48]. For example, overexpression of apple *SNAT5* increases melatonin biosynthesis, thereby conferring resistance to apple spot disease, whereas reduction in *SNAT5* via RNAi increases disease susceptibility by regulating the burst of extracellular reactive oxygen species [47]. Additionally, the increase in melatonin induced by alfalfa *SNAT1* expression is known to confer salt tolerance through the enhancement of antioxidant system enzyme activity and ion homeostasis [48]. Overexpression of Arabidopsis *SNAT1* exhibited resistance to UV-B stress by regulating the expression of several key components of the UV-B signaling pathway, such as the *bZIP transcription factor* and *UV-B photomorphogenesis repressor 1/2* (*RUP1/2*) [46]. Similarly, *CbSNAT* overexpression in rice resulted in high concentrations of melatonin in transgenic rice (CbSNAT OE lines) compared to the WT (Figure 5). This result supports that CbSNAT is implicated in melatonin biosynthesis. It is worth noting that the *K*_m_ value of CbSNAT toward 5-MT was 28 µM, the lowest *K*_m_ value detected among all SNAT enzymes (Table 1). This implies that CbSNAT can most efficiently use 5-MT for melatonin biosynthesis at lower substrate concentrations in cells. Moreover, the *K*_m_ value of CbSNAT toward serotonin (293 µM) was lower than that of other archaeal SNAT orthologs (Table 1).

Melatonin acts as a multifunctional biomolecule against various environmental stresses such as cold, heat, drought, and heavy metal stress [49]. In particular, melatonin is widely known to confer cadmium tolerance by inducing *ABC transporters* [50], *superoxide dismutase* [51], and *ascorbic acid peroxidase* [52]. In analogy, the melatonin-enriched CbSNAT OE lines exhibited tolerance to cadmium stress through the enhancement of chaperone genes such as *BIP3* and *BIP4*. In addition, the CbSNAT OE lines displayed improved resistance to the peroxidizing herbicide butafenacil, which inhibits PPO in the chlorophyll and heme biosynthetic pathways. PPO inhibition leads to an increase in the PPO enzymatic product proto IX rather than the PPO substrate protogen IX via a membrane-mediated oxidation pathway [53]. Proto IX accumulation results in light-dependent membrane damage through the rapid production of ROS.

Unlike cadmium tolerance, butafenacil tolerance in the CbSNAT OE lines was not attributed to the enhanced expression of chaperone genes such as *BIP3* and *BIP4*. Rather, *BIP3* and *BIP4* were downregulated in the CbSNAT OE lines relative to those of the WT following butafenacil treatment (Figure 6). Notably, *PPO1* expression was significantly enhanced in the CbSNAT OE lines compared to the WT against butafenacil treatment. Similar to CbSNAT OE, exogenous melatonin treatment induced butafenacil resistance along with increased *PPO1* expression. These results indicate that melatonin plays a protective role in butafenacil treatment by safeguarding chlorophyll and heme biosynthesis via the transcriptional regulatory effects of melatonin. Similar results were reported in melatonin-primed tomato seedlings exposed to heat stress; heat stress increased proto IX, which was prevented in unstressed melatonin-primed seedlings [54]. Additionally, pea seedlings exposed to the herbicide paraquat had increased proto IX levels following exogenous melatonin application, highlighting melatonin’s role in delaying chlorophyll degradation against oxidative stress [55].

**Figure 8:**
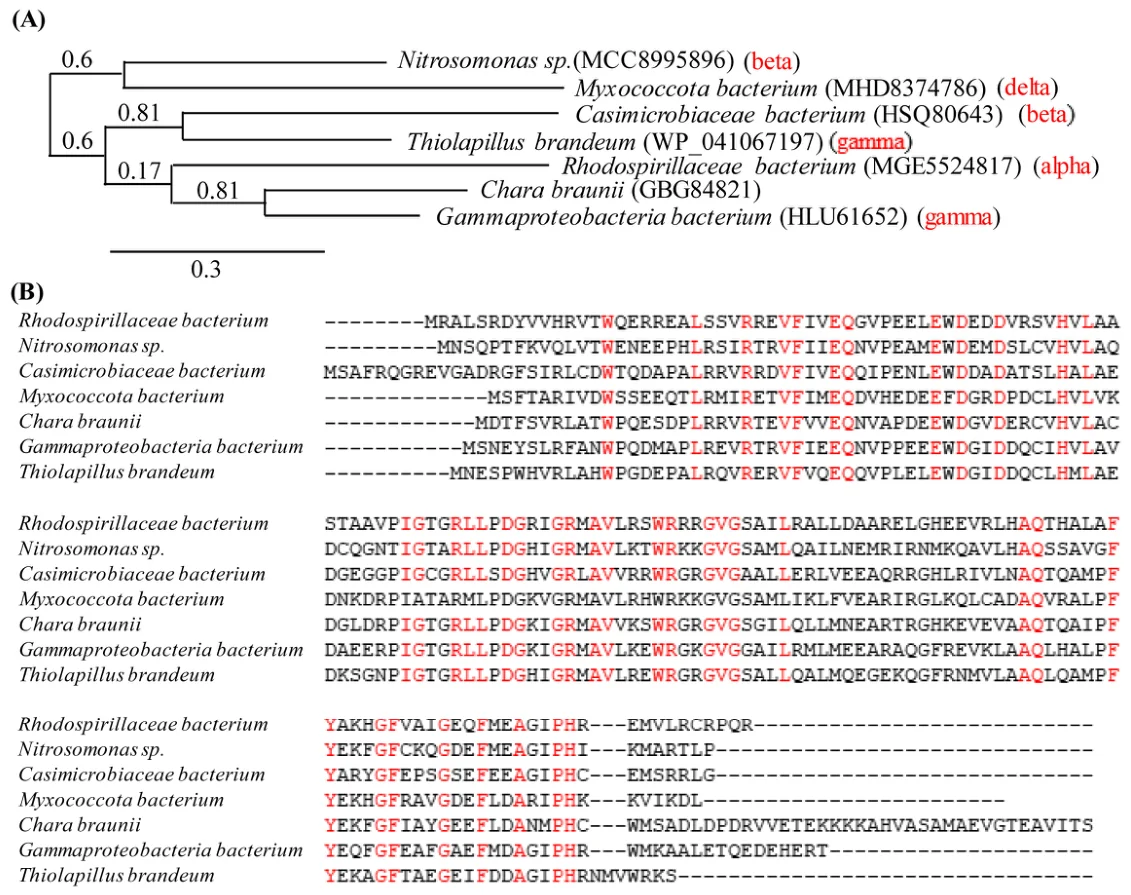
(**A**) Phylogenetic tree of SNAT proteins from proteobacteria genomes. The scale bar represents 0.3 substitutions per site. The GenBank access numbers are included in parentheses. Alpha, Beta, Gamma, and Delta refer to taxa of proteobacteria. (**B**) Amino acid sequence comparison among proteobacteria SNAT proteins. Dashes denote gaps. Conserved amino acids are denoted as red letters.

Many homologous proteins of CbSNAT are present in the genomes of Proteobacteria, but not in the genomes of Viridiplantae (Figure 8A). The *C. braunii* protein showed the highest aa identity of 64% to the Gammaproteobacteria GNAT family (GenBank accession number HLU61652), followed by 54% to *Nitrosomonas* sp. (Betaproteobacteria) GNAT (GenBank accession number MCC8995896), 53% to *Myxococcota bacterium* (Deltaproteobacteria) GNAT (GenBank accession number MHD8374786), and 53% to Rhodospirillaceae bacterium (Alphaproteobacteria) GNAT (GenBank accession number MGE5524817). However, there were no homologous proteins to CbSNAT in the genomes of Epsilonproteobacteria or Zetaproteobacteria. The Proteobacteria SNAT homolog proteins shared well-conserved consensus aa motifs with CbSNAT protein (Figure 8B).

**Figure 9:**
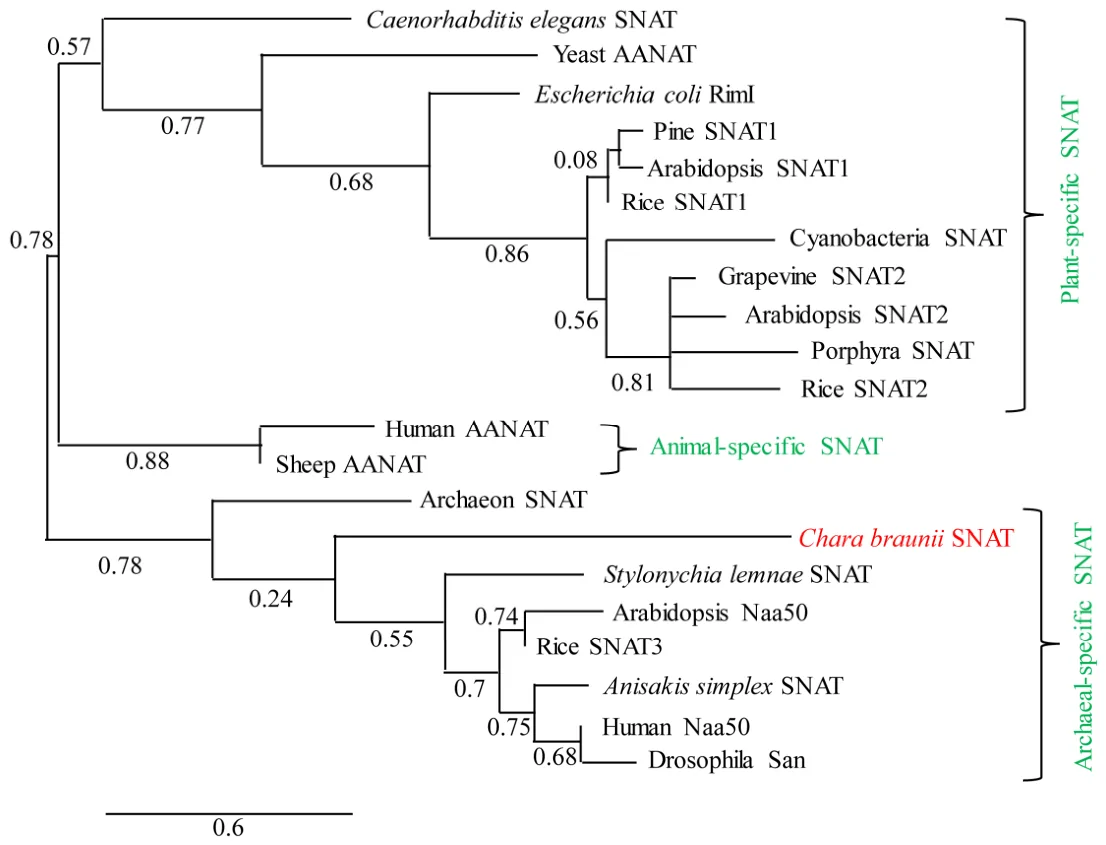
Phylogenetic tree of 21 SNAT proteins from 16 species. The scale bar represents 0.6 substitutions per site. GenBank accession numbers of 21 SNAT proteins were as follows: *Caenorhabditis elegans* SNAT1 (NM_065818); yeast AANAT (NP_010356); *E. coli* RimI (WP_137442509); pine SNAT1 (PSY00020345); Arabidopsis SNAT1 (BT024481); rice SNAT1 (AK059369); cyanobacteria SNAT (NP_442603); grapevine SNAT2 (RVX06207); Arabidopsis SNAT 2 (BT005218); porphyra SNAT (NC_007932); rice SNAT2 (AK068156); human AANAT (NP_001079); sheep AANAT (NP_001009461); archaeon SNAT (WP_010916271); *Chara braunii* SNAT (GBG84821); *Stylonychia lemnae* SNAT (CCKQ01002460); Arabidopsis Naa50 (NM_121172); rice SNAT3 (AK241100); *Anisakis simplex* SNAT (VDK51613); human Naa50 (BAB14397); Drosophila San (NM_080040).

Based on 21 full-length SNAT polypeptide sequences from 16 species, a phylogenetic tree was generated using the BLAST-Explorer program (version 2) (Figure 9). The resulting tree diagram resolved SNAT into three major clades: a plant clade, an animal clade, and an archaeal clade. The plant clade included various plant SNATs, *E. coli* RimI, yeast AANAT, and CeSNAT. In contrast, both human and sheep AANAT belonged to the animal clade. Notably, both the plant and animal clades evolved after the archaeal clade, which included *C. braunii* SNAT and human Naa50. Collectively, the archaeal SNAT clade appeared earlier than the plant and animal SNAT clades, acting as an ancestor *SNAT* gene for gene duplication into plant- and animal-specific *SNAT* genes. The fact that *C. braunii* SNAT homologs were predominantly found in members of the phylum Proteobacteria, including Alphaproteobacteria, suggests that archaeal SNATs originated earlier than plant- or animal-specific SNATs. The simultaneous existence of archaeal *SNAT*s and plant-specific *SNAT* homologues in the plant genome has the advantage of enabling effective and cooperative melatonin synthesis. Melatonin synthesized by multiple *SNAT* gene families not only acts directly as an antioxidant but also performs the role of a signaling molecule, influencing the expression of various antioxidant and defense-related genes against a wide range of stressors [1,8]. Therefore, since melatonin exhibits multiple resistances to various oxidative stresses, the results of this study are expected to provide useful information and practical techniques for breeding herbicide-resistant rice varieties through a melatonin-based DPE herbicide management approach different from the *PPO1* overexpression strategy. In other words, since melatonin acts as a sustainable and eco-friendly biostimulant, direct application to agriculture may be considered [56]. Further experiments are required to determine whether *PPO1* transcript induction in CbSNAT OE lines is directly associated with the induction of PPO enzyme activity and proto IX content.

## 5. Conclusions

The green alga *C. braunii* has an important bridging role in the evolutionary process from aquatic to terrestrial plants [57]. Although melatonin was detected and exogenous melatonin application improved the quantum yield of photosystem II in *C. australis* species [23], no melatonin biosynthesis genes such as *SNAT* have been reported due to the absence of SNAT homologs in the genomes of *Chara* species. In this study, we cloned a novel SNAT homolog from *C. braunii*, which showed a 28% aa identity to human Naa50, an archaeal SNAT ortholog. The purified recombinant CbSNAT proteins exhibited SNAT enzyme activity with high substrate affinity toward 5-MT and serotonin compared to other SNAT enzymes. Transgenic rice plants overexpressing *CbSNAT* produced more melatonin than did the non-transgenic WT, confirming that CbSNAT is functionally coupled to melatonin biosynthesis in rice. As is commonly observed in melatonin-enriched transgenic plants [37], the CbSNAT OE lines exhibited tolerance against the oxidative stressors cadmium and butafenacil. Butafenacil tolerance was attributable to the induction of the herbicide target gene *PPO1*. This report is the first to identify the interconnection between melatonin-mediated *PPO1* induction and butafenacil tolerance, which supports the potential of *CbSNAT* as a target gene to generate butafenacil-tolerant agricultural crops. Overall, *SNAT* is first cloned among the four melatonin biosynthetic genes present in *C. braunii* genome. *CbSNAT* will serve as a useful target gene for elucidating the detailed functions of melatonin by constructing transgenic *C. braunii* through the overexpression or deletion of *CbSNAT*. Furthermore, the cloning of *CbSNAT* from the *C. braunii* genome is expected to spark interest among other researchers in cloning studies of melatonin biosynthetic genes such as *TDC*, *T5H*, and *ASMT*.

## Figures and Tables

**Table 1:** Kinetic parameters of archaeon SNAT homologs (nd, not determined).

Species	Substrates	*K*_m_ (μM)	*V*_max_ (pmol/min/mg Protein)	*V*_max_/*K*_m_
*Thermoplasma volcanium*	Serotonin	621	416	0.669
	5-MT	nd	nd	nd
Human Naa50	Serotonin	986	1800	1.825
	5-MT	nd	nd	nd
*Escherichia coli* RimI	Serotonin	531	528	0.994
	5-MT	201	587	2.920
Rice SNAT3	Serotonin	1152	4800	4.166
	5-MT	1587	12,600	7.939
*Stylonychia lemnae*	Serotonin	776	1470	1.894
	5-MT	246	362	1.471
*Caenorhabditis elegans*	Serotonin	782	454	0.580
	5-MT	1413	1513	1.070
*Anisakis simplex*	Serotonin	408	960	2.352
	5-MT	188	690	3.670
*Chara braunii*	Serotonin	293	462	1.576
	5-MT	28	162	5.785

## Data Availability

The data presented in this study are available within the article and supplementary materials.

## Supplementary Materials

The following supporting information can be downloaded at: https://www.mdpi.com/article/10.3390/antiox15080942/s1, Figure S1: Butafenacil resistance in rice plants overexpressing *Stylonychia lemnae SNAT* (SlSNAT OE) and rice *SNAT3* (OsSNAT3 OE).
